# Anti-CD30 antibody–drug conjugate therapy in lymphoma: current knowledge, remaining controversies, and future perspectives

**DOI:** 10.1007/s00277-022-05054-9

**Published:** 2022-12-13

**Authors:** H. Miles Prince, Martin Hutchings, Eva Domingo-Domenech, Dennis A. Eichenauer, Ranjana Advani

**Affiliations:** 1 Sir Peter MacCallum Department of Oncology, The University of Melbourne, Melbourne, VIC Australia; 2 Epworth Healthcare, East Melbourne, VIC Australia; 3 Department of Haematology, Rigshospitalet, Copenhagen University Hospital, Copenhagen, Denmark; 4 Department of Hematology, Institut Catala D’Oncologia, Institut de Reserca IDIBELL, L’Hospitalet de Llobregat, Barcelona, Spain; 5 First Department of Internal Medicine, Center for Integrated Oncology Aachen Bonn Cologne Düsseldorf, University of Cologne, Cologne, Germany; 6 German Hodgkin Study Group (GHSG), First Department of Internal Medicine, University Hospital Cologne, Cologne, Germany; 7 Department of Medicine, Division of Oncology, Stanford University, Stanford, CA USA

**Keywords:** Classic Hodgkin lymphoma, Peripheral T-cell lymphoma, Cutaneous T-cell lymphoma, Antibody–drug conjugate, CD30 expression, Brentuximab vedotin

## Abstract

CD30 is overexpressed in several lymphoma types, including classic Hodgkin lymphoma (cHL), some peripheral T-cell lymphomas (PTCL), and some cutaneous T-cell lymphomas. The antibody–drug conjugate brentuximab vedotin targets CD30-positive cells and has been evaluated for the treatment of various lymphoma entities. This narrative review summarizes 10 years of experience with brentuximab vedotin for the treatment of CD30-positive lymphomas, discusses novel therapies targeting CD30 in development, and highlights remaining controversies relating to CD30-targeted therapy across lymphoma types. The collective body of evidence for brentuximab vedotin demonstrates that exploitation of CD30 can provide sustained benefits across a range of different CD30-positive lymphomas, in both clinical trials and real-world settings. Preliminary experience with brentuximab vedotin in combination with immune checkpoint inhibitors for relapsed/refractory cHL is encouraging, but further exploration is required. The optimal use of brentuximab vedotin for first-line therapy of PTCL remains to be determined. Further research is required on brentuximab vedotin treatment in high-risk patient populations, and in rare lymphoma subtypes, for which no standard of care exists. Novel therapies targeting CD30 include chimeric antigen receptor therapies and bispecific antibody T-cell engagers, which may be expected to further improve outcomes for patients with CD30-positive lymphomas in the coming years.

## Introduction

Several antigens that have limited expression in normal tissue and are overexpressed by tumor cells are excellent candidates for the development of immunotherapeutics. CD30, a member of the tumor necrosis factor receptor family, is expressed on a small subset of activated T and B lymphocytes and is overexpressed in a variety of lymphoma subtypes [1]. Highest CD30 expression is observed in classic Hodgkin lymphoma (cHL) and anaplastic large cell lymphoma (ALCL) [1]. Variable expression is observed across other lymphomas, including peripheral T-cell lymphomas (PTCL) [2], some cutaneous T-cell lymphomas (CTCL) such as mycosis fungoides (MF) [3], and some B-cell lymphomas such as diffuse large B-cell lymphoma (DLBCL) [4] (Table 1). CD30-positivity, typically measured by immunohistochemistry in tissue specimens, is defined as expression levels of ≥ 75% for ALCL; however, specific cut-offs for other tumor types are not universally defined, due to variability in expression levels [1]. Additionally, assay results can be variable due to the lack of a standardized diagnostic test.

**Table 1:** CD30 expression across PTCL and CTCL subtypes: data from key studies

*PTCL*	Proportion of patients with CD30 expression, % (n/total *n* of patients with lymphoma subtype)							
	**CD30 cut-off**	**PTCL-NOS**	**AITL**	**ATLL**	**ENKTL**	**ALK- ALCL**	**ALK+ ALCL**	**EATL**
Karube K et al. 2008 [122]	≥ 20%	11% (NS)	32% (NS)	24% (NS)	64% (NS)	58% (NS)		
Weisenburger DD et al. 2011 [123]	≥ 20%	32% (69/217)						
Sabattini E et al. 2013 [124]	≥ 25%	52% (45/87)	21% (9/42)		70% (7/10)			100% (9/9)
Bossard C et al. 2014 [125]	≥ 5%	58% (82/141)	63% (61/97)	56% (5/9)	46% (13/28)	100% (19/19)	100% (61/61)	50% (7/14)
Lamarque M et al. 2016 [126]	≥ 5%	90% (9/10)	100% (1/1)	0% (0/1)		100% (14/14)	56% (5/9)	100% (1/1)
Kawamoto K et al. 2018 [127]	≥ 25%				57% (55/97)			
Wang G-N et al. 2017 [128]	≥ 1%				70% (86/122)			
***CTCL***	**CD30 cut-off**	**CTCL**	**MF**	**T-MF**	**SS**			
Karube K et al. 2008 [122]	20–70%	9% (unknown)						
Sabattini E et al. 2013 [124]	≥ 25%		13% (4/32)	100% (9/9)				
Benner MF et al. 2012 [129]	> 25%			47% (47/100)				
Klemke CD et al. 2015 [130]	≥ 5%				33% (11/49)			
**B-cell lymphomas**	**CD30 cut-off**	**DLBCL**						
Salas MQ et al. 2020 [114]	> 20%	19% (41/216)						
Rodrigues-Fernandes CI et al. [4]	> 20%	2.5–37% (NS)						
Slack GW et al. 2014 [131]	≥ 20%	12% (47/385)						
Hu S et al. 2013 [132]	≥ 20%	14% (65/461)						

AITL, angioimmunoblastic T-cell lymphoma; ALCL, anaplastic large cell lymphoma; ALK, anaplastic lymphoma kinase; ATLL, adult T-cell leukemia/lymphoma; CTCL, cutaneous T‐cell lymphoma; DLBCL, diffuse large B-cell lymphoma; EATL, enteropathy-associated T-cell lymphoma; ENKTL, extranodal natural killer/T-cell lymphoma; MF, mycosis fungoides; NS, not stated; PTCL, peripheral T-cell lymphoma; PTCL-NOS, PTCL not otherwise specified; SS, Sézary syndrome; T-MF, transformed MF

Despite the viability of CD30 as a therapeutic target, initial clinical trials with “naked” anti-CD30 antibodies yielded disappointing clinical responses [1, 5]. The subsequently developed antibody–drug conjugate (ADC) brentuximab vedotin, consisting of a humanized IgG1 anti-CD30 monoclonal antibody conjugated to the antimitotic agent monomethylauristatin E (MMAE) by a cleavable linker, has proven to be substantially effective [5–7]. Brentuximab vedotin acts by binding of the antibody component to CD30-positive cells, followed by endocytosis and release of MMAE upon exposure to intracellular lysozymes, and inhibition of tubulin formation resulting in cell apoptosis [5–7]. Once MMAE is released, it may induce further tumor cell death by diffusion across the cell membrane to penetrate neighboring cells, an effect known as “bystander killing” [8]. Brentuximab vedotin was first approved for treatment of relapsed/refractory cHL and ALCL in the USA in 2011 and Europe in 2012 [6, 7].

In this review, we describe current evidence for anti-CD30 therapy in lymphoma, discuss ongoing controversies and data gaps, and provide perspectives on the direction of anti-CD30 therapy in the future.

## CD30-targeted therapy for cHL

The development of brentuximab vedotin has altered the treatment approach for patients with cHL. Treatment indications have been expanded since the initial approvals (Table 2). National Comprehensive Cancer Network^®^ (NCCN^®^) Clinical Practice Guidelines In Oncology (NCCN Guidelines^®^) recommend brentuximab vedotin in combination with doxorubicin, vinblastine, and dacarbazine (AVD) as a primary treatment option for stage III–IV disease, and as single-agent maintenance therapy following high-dose therapy and autologous stem cell transplantation (ASCT) for refractory disease with high risk of relapse [9]. European Society for Medical Oncology guidelines recommend brentuximab vedotin as consolidation treatment following high-dose chemotherapy and ASCT in patients with poor-risk factors, and as possible salvage therapy before high-dose chemotherapy and ASCT [10]. In older adults > 60 years of age, brentuximab vedotin is recommended by NCCN as a primary treatment option for stage III–IV disease in combination with AVD or dacarbazine [9]. The NCCN Guidelines^®^ for pediatric HL recommend brentuximab vedotin in combination with bendamustine, gemcitabine, or nivolumab as a reinduction therapy option for patients with relapsed/refractory cHL, and state that it may also be useful as maintenance post-transplant for some high-risk pediatric patients [11].

**Table 2:** Brentuximab vedotin indications in the USA and Europe [6, 7]

USA	Europe
***HL***	
Adults with previously untreated stage III or IV cHL in combination with chemotherapy	Adult patients with previously untreated CD30+ stage IV HL in combination with AVD
Adults with cHL at high risk of relapse or progression as post-auto-HSCT consolidation	Adult patients with CD30+ HL at increased risk of relapse or progression following ASCT
Adults with cHL after failure of auto-HSCT or after failure of at least 2 prior multi-agent chemotherapy regimens in patients who are not auto-HSCT candidates	Adult patients with relapsed or refractory CD30+ HL following ASCT, or following at least 2 prior therapies when ASCT or multi-agent chemotherapy is not a treatment option
***PTCL***	
Adults with sALCL after failure of at least 1 prior multi-agent chemotherapy regimen	Adult patients with previously untreated sALCL in combination with CHP
	Adult patients with relapsed or refractory sALCL
***CTCL***	
Adults with C-ALCL or CD30-expressing MF who have received prior systemic therapy	Adult patients with CD30+ CTCL after at least 1 prior systemic therapy

ASCT, autologous stem cell transplant; auto-HSCT, autologous hematopoietic stem cell transplant; AVD, doxorubicin, vinblastine and dacarbazine; C-ALCL, primary cutaneous anaplastic large cell lymphoma; cHL, classical Hodgkin lymphoma; CHP, cyclophosphamide, doxorubicin, and prednisone; CTCL, cutaneous T-cell lymphoma; HL, Hodgkin lymphoma; MF, mycosis fungoides; PTCL, peripheral T-cell lymphoma; sALCL, systemic anaplastic large cell lymphoma

## Brentuximab vedotin for first-line treatment of cHL

The recommendation for brentuximab vedotin in combination with AVD as primary treatment for cHL is based on data from the phase III ECHELON-1 study. In this trial, patients with previously untreated stage III–IV disease were randomized to receive brentuximab vedotin plus AVD (A + AVD) or bleomycin plus AVD (ABVD) [12]. The primary endpoint of modified progression-free survival (mPFS), defined as time to disease progression, death or evidence of noncomplete response followed by subsequent anticancer therapy, was significantly higher in the A + AVD arm versus the ABVD arm (2-year mPFS per independent review 82.1% vs. 77.2%, hazard ratio (HR) 0.77, 95% confidence interval (CI) 0.60–0.98; *p* = 0.03) [12]. Secondary endpoints also trended in favor of A + AVD [12]. The benefit of A + AVD was sustained at a median follow-up of 60.9 months, with a 5-year PFS per investigator of 82.2% in the A + AVD arm compared with 75.3% in the ABVD arm (HR 0.681, 95% CI 0.534–0.867; *p* = 0.002) [13]. Five-year PFS per investigator was consistently higher with A + AVD versus ABVD across age groups and those with positive or negative positron emission tomography (PET) status after cycle 2 [13]. Given that, historically, the majority of relapses in cHL occur within the first 5 years after completion of first-line treatment [14], these data suggest that more patients receiving A + AVD achieved long-term remission or were cured of their disease, compared with those receiving ABVD. Consistent with this, a significant improvement in overall survival (OS) and PFS with A + AVD versus ABVD in ECHELON-1 (OS: HR 0.59, 95% CI 0.396–0.879, *p* = 0.009; PFS: HR 0.68, 95% CI 0.53–0.86; *p* = 0.002) after a median of approximately 6 years follow-up has recently been reported [15]. A + AVD is therefore an attractive treatment option for this patient group, especially given the generally manageable safety profile. Although peripheral neuropathy (PN) occurred in a higher proportion of patients in the A + AVD arm than the ABVD arm in the primary analysis (67% vs. 43%), by the 6-year analysis, most PN had resolved, with 18.9% and 9% of patients, respectively, having ongoing PN, most of which were grade 1/2 [15]. Of those with PN in the A + AVD arm in the primary analysis, 71.8% had complete resolution and 13.8% had improved at 6 years [15]. Febrile neutropenia also occurred at a higher rate in the A + AVD arm [12, 15]; however, this appeared to be manageable with granulocyte-colony stimulating factor (G-CSF) support, with a lower incidence in patients who received primary prophylaxis with G-CSF compared with those who did not [12, 15, 16]. These data support the use of G-CSF primary prophylaxis in all patients who receive A + AVD as first-line treatment for stage III–IV cHL [16]. A + AVD was also associated with numerically fewer second primary malignancies compared with ABVD (23 [14 solid and 9 hematological] vs. 32 [14 solid and 17 hematological]), as well as a greater number of pregnancies (114 vs. 81), suggesting no additional risk of infertility [15].

Subgroup analyses from ECHELON-1 assessed the efficacy and safety of A + AVD as first-line treatment in older and high-risk patients [17, 18]. In adults ≥ 60 years of age, a group for whom outcomes have historically been poor, modified PFS at 24 months was statistically similar between the A + AVD and ABVD arms [17]. The 5-year follow-up analyses revealed a numerical improvement in PFS per investigator favoring A + AVD over ABVD in patients aged ≥ 60 years [13]. A + AVD was associated with a higher incidence of PN and neutropenia, but a lower incidence of pulmonary-related toxicity compared with ABVD [17]. In patients with stage IV disease or International Prognostic Score (IPS) of 4–7, who have a comparatively lower 5-year failure-free survival rate compared with patients with less advanced disease, benefits of A + AVD versus ABVD in relation to PFS were apparent at a median follow-up of 3 years (stage IV disease HR 0.723, 95% CI 0.537–0.973; *p* = 0.032; IPS 4–7, HR 0.588, 95% CI 0.386–0.894; *p* = 0.012) [18]. Patients in these high-risk groups experienced similar incidence and severity of adverse events (AEs) to the overall ECHELON-1 safety population [18].

Other brentuximab vedotin combinations with chemotherapy have also been evaluated for first-line treatment of adults with cHL. In a phase II trial of patients with newly diagnosed advanced-stage cHL, brentuximab vedotin in combination with etoposide, cyclophosphamide, doxorubicin, and either procarbazine and prednisone (BrECAPP) or dacarbazine and dexamethasone (BrECADD) provided promising 3-year PFS rates of 90.2% and 89.7%, respectively [19]. The BrECADD combination, in particular, was associated with a more favorable toxicity profile in comparison to the standard escalated bleomycin, etoposide, doxorubicin, cyclophosphamide, vincristine, procarbazine, and prednisone (BEACOPP) regimen [20]. A randomized phase III study (HD21) of BrECADD compared with escalated BEACOPP has completed accrual. The upcoming results of this study will answer the question of whether BrECADD can replace interim PET-guided escalated BEACOPP (4 cycles in case of a negative PET after 2 cycles; 6 cycles in case of a positive PET after 2 cycles) as the standard-of-care treatment for advanced cHL in countries in which this approach is used [21]. Another study of brentuximab vedotin in first-line therapy of advanced-stage cHL has also completed accrual: the phase II COBRA study used A+AVD as the initial therapy for all patients, with an early interim PET/CT following cycle 1 [22]. In case of a positive PET result, the patients shifted from A+AVD to BrECADD, with the goal to reach similar efficacy as in BrECADD-treated cohorts but reserving the more intensive treatment to those with suboptimal initial response.

In previously untreated patients for whom conventional chemotherapy is not an option due to age, frailty, or comorbidities, brentuximab vedotin monotherapy has been shown to be tolerable but with limited activity, highlighting a need for research into combination with other agents in this patient population [23]. A phase II study comprising 48 patients with cHL aged ≥ 60 has investigated sequential brentuximab vedotin before and after chemotherapy with AVD. Treatment was well tolerated in most patients and 2-year outcomes were promising with PFS and OS rates of 84% and 93%, respectively [24].

Most patients with limited-stage HL are cured with standard therapy, but some have reduced life-spans due to increased risk of late radiotherapy-related complications, such as malignancies [25] (although this has become less common in the era of involved-site radiation therapy). In a phase II study in which patients with previously untreated non-bulky stage I–II cHL received brentuximab vedotin as consolidation therapy after chemotherapy with ABVD, estimated 3-year PFS and OS were 92% and 97%, respectively, and median PFS and OS were not reached at a median follow-up of 47 months, suggesting encouraging activity in this population [25]. In the randomized phase II BREACH study, which compared A+AVD with ABVD in early unfavorable HL, more patients in the A + AVD group versus the ABVD group were PET-negative after 2 treatment cycles (82.3% vs. 75.4%) [26].

Published data on first-line brentuximab vedotin combined with chemotherapy in patients aged ≤ 18 years are limited. In a single-arm multicenter trial in pediatric patients with previously untreated stage IIB–IV cHL, brentuximab vedotin was used in place of vincristine in an OEPA/COPDac regimen (vincristine, etoposide, prednisone, and doxorubicin/cyclophosphamide, vincristine, prednisone, and dacarbazine) [27]. After a median follow-up of 3.4 years, 3-year event-free survival (EFS) was 97.4%, and OS was 98.7%, and brentuximab vedotin was well tolerated, supporting evaluation of this regimen in pediatric patients in a randomized controlled trial setting [27]. In the phase I/II (NCT02979522) clinical trial, which assessed the safety and activity of first-line A + AVD in pediatric patients with advanced stage cHL, overall response rate (ORR) and complete response (CR) rate at the end of treatment were 88% and 76%, respectively [28].

## Brentuximab vedotin for relapsed/refractory HL following ASCT

In an early phase II study that investigated brentuximab vedotin monotherapy in patients who had already relapsed or were refractory post-ASCT, brentuximab vedotin induced objective responses in 75% of patients [29]. A recent meta-analysis showed that brentuximab vedotin treatment post-ASCT, either as monotherapy or part of a salvage chemotherapy regimen, has long-term benefits, with 5-year PFS and OS of 31% and 34%, respectively [30]. Reports on brentuximab vedotin treatment post-allogeneic stem cell transplantation (SCT) suggest that brentuximab vedotin provides transient control of cHL with 60–70% of patients achieving ORRs lasting a median of 6–7 months and a median PFS in the region of 7 months [31–33].

## Brentuximab vedotin for relapsed/refractory cHL

A phase II trial of brentuximab vedotin as second-line monotherapy in patients with relapsed/refractory cHL reported an ORR of 75% and CR rate of 43%, representing an effective bridge to ASCT [34]. Brentuximab vedotin has also been assessed as a bridge to allogeneic SCT in relapsed/refractory cHL [35–37]. Various brentuximab vedotin plus chemotherapy combinations have been investigated as salvage for patients with relapsed/refractory disease in clinical trials [38–41] and have previously been reviewed [42]. Combinations including brentuximab vedotin plus bendamustine; brentuximab vedotin combined with ifosfamide, carboplatin, and etoposide (A-ICE); brentuximab vedotin plus dexamethasone; high-dose cytarabine and cisplatin (BV-DHAP); and brentuximab vedotin combined with etoposide, methylprednisolone, high-dose cytarabine, and cisplatin (BrESHAP) have all resulted in high pre-ASCT CR rates, and encouraging preliminary PFS and OS [38–41].

Treatment regimens combining brentuximab vedotin with immunotherapeutic agents have also been evaluated in the relapsed/refractory setting, although data are currently limited to early-phase studies. There is a strong rationale for such combinations, as these therapies have distinct mechanisms of action. Immune checkpoint inhibitors facilitate reactivation of T-cells that can target Hodgkin and Reed-Sternberg cells and may overcome the negative impact of anti-programmed cell death-1 (PD-1) pathway derangement caused by gene amplification [43]. Brentuximab vedotin complements this by disrupting the microtubule network within CD30-expressing tumor cells leading to apoptosis [7], while also having immunomodulatory activity through induction of immunogenic cell death resulting in activation of innate and adaptive immune cells [44–46]. Immune cell activation coupled with checkpoint blockade may be a promising therapeutic approach in cHL. A phase I/II study of brentuximab vedotin plus nivolumab as first salvage therapy showed durable efficacy at a median follow-up of 34.3 months, with ORR of 85%, CR rate of 67%, and 3-year PFS and OS of 77% and 93%, respectively [47]. A trial of brentuximab vedotin as triplet therapy with ipilimumab and nivolumab suggested responses similar to or greater than that of brentuximab vedotin plus nivolumab and greater than that of brentuximab vedotin plus ipilimumab; this is now being evaluated in a randomized trial [48]. No clinical trial data are available for the combination of brentuximab vedotin plus pembrolizumab, although a durable response with this combination has been noted in a single case report [49]. Recent data from the KEYNOTE-204 phase III study suggest that pembrolizumab monotherapy leads to significantly improved PFS compared with brentuximab vedotin monotherapy in patients with relapsed/refractory cHL who were ineligible for or relapsed after ASCT, with median PFS of 13.2 months for pembrolizumab versus 8.3 months for brentuximab vedotin after 25.7 months follow-up [50]. Nevertheless, dual therapy with checkpoint inhibitors and brentuximab vedotin may have additional benefits and is under investigation.

## Brentuximab vedotin maintenance following ASCT

The phase III AETHERA trial enrolled adults with unfavorable-risk cHL who had undergone HDT and ASCT and were at high risk of relapse or progression due to primary refractory HL, relapsed HL with remission duration of < 12 months, or extranodal involvement at the start of pre-transplantation salvage chemotherapy [51]. PFS was significantly improved in patients who received brentuximab vedotin versus those who received placebo after a median observation time of 30 months (HR 0.57, 95% CI 0.40–0.81; *p* = 0.0013); however, no significant difference in OS was seen [51]. This benefit was sustained at 5 years (HR 0.521, 95% CI 0.379–0.717), and a considerable delay in time to second subsequent anticancer therapy with brentuximab vedotin was also observed [52]. Treatment with brentuximab vedotin was generally well tolerated with a safety profile that was consistent with previous experience with the drug; PN was the most common AE with brentuximab vedotin, but most events were mild to moderate in severity and the majority resolved or improved during observation [51, 52].

## Real-world data on brentuximab vedotin in cHL

Real-world data on brentuximab vedotin for cHL have demonstrated the utility and feasibility of CD30-targeted ADC therapy beyond the setting of clinical trials and specialized centers (Table 3). Although there was a paucity of data on the real-world use of brentuximab vedotin as consolidation therapy post-ASCT for some time, several recent retrospective studies have assessed the effectiveness of this approach in adults, children, and adolescents, and support the results of the AETHERA trial [53–57]. Real-world data also support the efficacy and acceptable safety profile of brentuximab vedotin in adults with refractory or relapsed cHL [58, 59], including as a bridge to ASCT [60], and the use of brentuximab vedotin in combination with bendamustine in children, adolescents, and adults with cHL [61, 62].

**Table 3:** Key real-world evidence on brentuximab vedotin for the treatment of cHL

Publication	Country	*N*	Median follow-up (months)	Prior brentuximab vedotin salvage, %	≥ 2 Risk Factors, %	Complete response pre-ASCT, %	Median brentuximab vedotin cycles	PFS / OS, %	Key risk factors
**Consolidation therapy post-ASCT (adults)**									
Massaro M et al. [53]	Italy	105	20	51	29	75	10	3Y: 62/86	• Refractory disease (48%) • CR < 12 months (49%) • Extranodal disease at relapse (22%)
Marouf A et al. [55]	France	115	35	70	NR	81	11	2Y: 75/96	• Primary refractory disease (43%) • Early relapse (27%) • Extranodal disease (49%)
Akay OM et al. [56]	Turkey	75	26	23	NR	43	NR (8 in those who discontinued)	2Y: 68/88	• Primary refractory or relapse < 12 months (*n* = 61) • No CR to last salvage regimen (*n* = 51) • ≥ 2 salvage regimens (*n* = 29)
Chung S et al. [54]	Canada	114	62	9	NR	NR	10.5	5Y: 56/86	• Primary refractory cHL (40%) • Relapse < 12 months (18%) • Extranodal disease at relapse (13%)
**Consolidation therapy post-ASCT (children/adolescents)**									
Forlenza C et al. [57]	USA/Canada	52	34	81	71	77	12	3Y: 92/96	• Refractory or relapse < 12 months • Extranodal disease • B symptoms • Best response PR/SD to salvage therapy
**Bridge to ASCT (adults)**									
Pinczes LI et al. [60]	Hungary	41	17	NA	NR	71	3	2Y: 62/93	• Relapsed or refractory cHL • Stage III–IV disease (56%) • Extranodal disease (20%)
**Salvage therapy for relapsed or refractory disease (adults)**									
Kral Z et al. [59]	Czech Republic/Slovakia	58	17	NR	NR	NA	7.5	2Y: 45/3Y: 41	• Relapse after ASCT (91%) • Relapsed/refractory and unsuitable for ASCT (9%)
Izutsu K et al. [58]	Japan	182	9	NR	NR	NR	5.5	1Y: NR/83	• Prior chemotherapy (98%) • Stage IV disease (47%) • Prior ASCT (18%)
Uncu-Ulu B et al. [62]	Turkey	61	14	52	NR	NR	4	2Y: 60/72	• Primary refractory disease (47.5%) • Relapse (52.5%)
**Salvage therapy for relapsed or refractory disease (children/adolescents)**									
McMillan A et al. [61]	UK	29	24	10	NR	79	3	2Y: 64/90	• Primary refractory disease (69%) • Early relapse (7%) • Late relapse (24%)

ASCT, autologous stem cell transplant; cHL, classical Hodgkin lymphoma; CR, complete response; NA, not applicable; NR, not reported; OS, overall survival; PFS, progression-free survival; PR, partial response; SD, stable disease; Y, year

## Considerations for brentuximab vedotin rechallenge

Despite excellent response rates and acceptable tolerability, most patients will eventually progress on single-agent brentuximab vedotin therapy [63]. It has been hypothesized that such patients may benefit from a second course of brentuximab vedotin treatment. In a phase II study of cHL patients who previously achieved an objective response with brentuximab vedotin therapy in a clinical trial and who received a second course of brentuximab vedotin treatment, ORR was 60%, and CR rate was 30% [64]. These results were borne out by a retrospective study of brentuximab vedotin-retreated patients in which ORR was 52.9%, CR was 17.6%, and median PFS was 5.3 months [65]. Retreatment with brentuximab vedotin may therefore be a viable alternative to serial palliative chemotherapy regimens [64].

## Novel anti-CD30 therapies for relapsed/refractory HL

Experience with brentuximab vedotin has demonstrated the benefits of targeting CD30 in relapsed/refractory cHL, paving the way for development of new CD30-based therapies. An initial phase I study targeting CD30 with T-cells expressing a CD30-specific chimeric antigen receptor (CAR-T) reported good tolerability but limited clinical responses in 6 patients with relapsed or refractory cHL (of whom 5 had previously received brentuximab vedotin), with an ORR of 33% [66]. Efficacy was improved in a subsequent phase I/II study of CD30 CAR-T cells in combination with lymphodepleting chemotherapy, in which the ORR was 62% and 1-year PFS and OS were 36% and 94%, respectively [67]. These results were confirmed in the phase II CHARIOT study of patients with cHL who progressed after ≥ 3 lines of therapy. Preliminary data from the pilot segment showed that autologous CD30 CAR-T cells with lymphodepleting chemotherapy resulted in an ORR of 100% (5/5 patients) and CR rate of 80% (4/5 patients) by day 42, with a well-tolerated safety profile [68, 69]. A case study has suggested that the addition of a second target, such as CD19, to CAR-T cells may also result in improved responses [70]. Thus, CAR-T cells represent an important new therapeutic option for relapsed/refractory cHL that may improve patient outcomes in the future.

Bispecific T-cell engagers are another type of novel CD30-based therapy in clinical development. The CD30/CD16A-bispecific antibody AFM13 is a first-in-class innate cell engager that binds to CD16A on innate immune cells and CD30 on cHL cells, resulting in recruitment and activation of innate immune cells in close proximity to tumor cells, facilitating antibody-dependent cell-mediated cytotoxicity (ADCC) [71, 72]. Activity of AFM13 was confirmed in a phase I study [71]. However, a subsequent phase II study has only shown modest activity [73]. The combination of AFM13 with pembrolizumab resulted in a high ORR of 83% in a phase Ib study [72], and AFM13 in combination with cytokine-activated adult blood or cord-blood natural killer (NK) cells has also been highlighted as a promising combination in preclinical models [74]. A CD30/CD137 bispecific antibody has also been developed [75]. Like CD30, CD137 is also frequently expressed on malignant cells in cHL; approximately 86% of cHL cases have CD137-expressing cells [75].

## Ongoing controversies for anti-CD30 therapy in cHL

Despite the wealth of clinical evidence for brentuximab vedotin therapy in cHL, a number of questions remain. Firstly, the combination of brentuximab vedotin with immune checkpoint inhibitors in relapsed/refractory cHL has great potential, but it remains to be seen whether such combinations provide greater benefits than brentuximab vedotin plus chemotherapy or immune checkpoint inhibitors plus chemotherapy (e.g., pembrolizumab plus gemcitabine, vinorelbine, and doxorubicin [76]). Cost-effectiveness assessments to determine whether demonstrated benefits justify the increase in cost of treatment with brentuximab vedotin and immune checkpoint inhibitors over chemotherapy-based regimens will also be important.

Secondly, for first-line treatment of advanced cHL, further data are required to determine the optimal brentuximab vedotin combination regimen. An ongoing phase III study comparing A + AVD versus nivolumab plus AVD in patients with advanced cHL will assess the value of first-line brentuximab vedotin and immune checkpoint inhibitor combination therapy [77].

Thirdly, the use of PET status after cycle 2 (PET2) to guide potential switching of first-line therapy in patients with cHL remains unclear. Results from ECHELON-1 found that while PET2-positive and PET2-negative subgroups both benefitted from A + AVD compared with ABVD in terms of 5-year PFS per investigator, PET2-negative patients still fared better than PET2-positive patients; 5-year PFS rates for PET2-negative patients were 84.9% (95% CI 81.7–87.6) with A + AVD and 78.9% (95% CI 75.2–82.1) with ABVD, while in PET2-positive patients, PFS rates were 60.6% (95% CI 45.0–73.1) with A + AVD and 45.9% (95% CI 32.7–58.2) with ABVD [13]. This suggests that there may still be room for improvement in the treatment of patients who remain PET2-positive.

Finally, there is a scarcity of data from randomized controlled trials assessing the efficacy of brentuximab vedotin retreatment following first-line brentuximab vedotin in the relapsed/refractory cHL setting. Data from the ECHELON-2 study, which assessed the efficacy and safety of first-line brentuximab vedotin in patients with PTCL, suggest that retreatment of patients with cHL with brentuximab vedotin may be worth investigating [78].

## CD30-targeted therapy for PTCL

CD30 is consistently expressed by systemic ALCL (sALCL) cells and is an established component of sALCL diagnosis [2]. As such, brentuximab vedotin plus cyclophosphamide, doxorubicin, and prednisone (CHP) is indicated for previously untreated sALCL [7], and as a single agent for relapsed/refractory sALCL [6, 7]. Additionally, recent studies indicate a role for CD30 in other PTCL subtypes. CD30 is not always universally expressed on all PTCL cells, but has a prevalence of around 32–64% in PTCL not otherwise specified (PTCL-NOS), 50–100% in enteropathy-associated T-cell lymphoma (EATL), and 46–80% in angioimmunoblastic T-cell lymphoma (AITL) [2]. International guidelines recommend brentuximab vedotin as a preferred choice of second-line therapy for sALCL [79]. NCCN Guidelines recommend brentuximab vedotin plus CHP as a preferred first-line therapy option for ALCL (category 1) or other CD30-positive entities including PTCL-NOS (category 2A). NCCN Guidelines also recommend brentuximab vedotin as the preferred choice of second-line therapy for relapsed/refractory ALCL and other CD30-positive entities, including PTCL-NOS [80].

An early clinical trial in which patients with CD30-positive PTCL received brentuximab vedotin administered sequentially with cyclophosphamide, doxorubicin, vincristine, and prednisone (CHOP) or in combination with CHP and reported CR rates of 62% and 88% and 1-year PFS of 77% and 71%, respectively [81]. Based on these encouraging results, the global phase III ECHELON-2 study was performed, in which newly diagnosed patients with CD30-positive PTCL were randomized to receive brentuximab vedotin plus CHP or CHOP [82]. By design, patients with sALCL made up 70% of the study population, while patients with PTCL-NOS, AITL, adult T-cell leukemia/lymphoma (ATLL), and EATL made up the remainder [82]. CD30-positivity was defined as ≥ 10% CD30-positive cells. PFS and OS were significantly greater with brentuximab vedotin plus CHP compared with CHOP at the primary analysis cut-off (PFS HR 0.71, 95% CI 0.54–0.93; *p* = 0.0110; OS HR 0.66, 95% CI 0.46–0.95, *p* = 0.0244), with median PFS of 48 months versus 21 months after a median follow-up of 36 months [82]. Patients with ALCL kinase (ALK)-positive and ALK-negative sALCL had the lowest HR point estimate for PFS of 0.29 (0.11–0.79) and 0.65 (0.44–0.95), respectively, indicating a clear benefit of brentuximab vedotin plus CHP. However, the HR estimate for PTCL-NOS was 0.75 (0.41–1.37) and AITL was 1.4 (0.64–3.07) and, therefore, not statistically significant. One would interpret this data to indicate that there was no benefit demonstrated in this study for adding brentuximab vedotin for patients with AITL. However, there were only 54 patients with AITL in the study and more research is required for this group of patients. Similarly, for patients with PTCL-NOS (n = 62 in the study) there was only a trend for benefit demonstrated; again, more research is required particularly examining molecular subgroups of PTCL-NOS [82]. The safety profile of brentuximab vedotin plus CHP was manageable, with a similar incidence of AEs to the CHOP arm, including febrile neutropenia and PN. The incidence and severity of neutropenia was lower in patients who received primary prophylaxis with G-CSF [82]. A subsequent 5-year analysis of ECHELON-2 demonstrated continued benefits of brentuximab vedotin plus CHP versus CHOP after a median follow-up of 47.6 months (PFS HR 0.70, 95% CI 0.53–0.91; *p* = 0.0077; OS HR 0.72, 95% CI 0.53–0.99; *p* = 0.0424), which was most notable in the subgroup of patients with sALCL [78]. Furthermore, a phase II study investigating the efficacy and safety of brentuximab vedotin plus CHP in front-line treatment of patients with PTLC with < 10% CD30-positive cells is ongoing (NCT04569032) [83].

In a phase II trial of 46 patients with newly diagnosed PTCL (including 16 with ALCL, 18 with AITL and 11 with PTCL-NOS), the addition of etoposide to brentuximab vedotin plus CHP followed by brentuximab vedotin consolidation resulted in an ORR of 91% and a CR rate of 80%, with 18-month PFS and OS of 61% and 89%, respectively [84]. Another phase II study of brentuximab vedotin plus CHP followed by HDT and ASCT for first-line treatment of patients with EATL demonstrated an ORR of 79% and a CR rate of 64%, with 2-year PFS and OS of 63% and 68%, respectively, after a median follow-up of 2.1 years [85].

Brentuximab vedotin has been evaluated for the treatment of pediatric patients with newly diagnosed ALK-positive CD30-positive ALCL [86]. The addition of brentuximab vedotin to 6 alternating cycles of chemotherapy, consisting of methotrexate, dexamethasone, ifosfamide, etoposide, and cytarabine (cycles 1, 3, and 5) and methotrexate, dexamethasone, cyclophosphamide, and doxorubicin (cycles 2, 4, and 6), in these patients did not add significant toxicity and resulted in 2-year EFS and OS of 79.1% and 97.0%, respectively [86].

## Real-world data on brentuximab vedotin in PTCL

Aside from ECHELON-2, clinical trial data on brentuximab vedotin therapy for PTCL are limited. However, several analyses have assessed brentuximab vedotin in patients with PTCL in real-life settings. In a single-center study of 27 patients with refractory/relapsed PTCL treated with brentuximab vedotin in a real-life practice, ORR was 63.0%, and after a median follow-up of 12.5 months, median PFS and OS were 5.2 and 12.5 months, respectively [87]. Response rates were improved when brentuximab vedotin was used in combination with chemotherapy compared with brentuximab vedotin monotherapy [87]. In 101 patients with sALCL in Japan, brentuximab vedotin treatment led to a 1-year OS of 79.3% and an ORR of 62.0% [58]. In 52 patients with sALCL who received brentuximab vedotin at relapse (primarily as second-line therapy), 3-year OS was 40.2% (median follow-up 34 months) [88]. In a retrospective study of brentuximab vedotin in combination with ICE for relapsed or refractory PTCL, 4 of 14 patients achieved an overall response and 2 achieved CR, with 2-year PFS and OS of 14% and 17.5%, respectively [89]. A retrospective study of brentuximab vedotin plus bendamustine in patients with relapsed/refractory PTCL demonstrated an ORR of 71% and a CR rate of 51% and median PFS and OS of 8.3 and 26.3 months, respectively, with a median follow-up of 9 months [90].

## Novel anti-CD30 therapies for relapsed/refractory PTCL

Currently, research into new anti-CD30 therapies for the treatment of PTCL appears to be limited. CD30 CAR-T cells have been shown to suppress tumor growth and increase tumor cell lysis in preclinical PTCL models [91], and led to durable remission in a single patient with EATL that was relapsed/refractory to multiple lines of chemotherapy, brentuximab vedotin, and ASCT [92]. Further studies are required to investigate the efficacy of CAR-T cells across subtypes of PTCL. A phase II study to assess the efficacy and safety of the CD30/CD16A-bispecific antibody AFM13 in patients with CD30-positive PTCL is ongoing [93].

## Ongoing controversies for anti-CD30 therapy in PTCL

As the clinical efficacy of brentuximab vedotin is lower in PTCL than cHL, there are numerous outstanding questions. It is not clear whether brentuximab vedotin therapy is appropriate for use upfront in all PTCL cases, especially given the variability in CD30 expression across subtypes. Some studies have indicated that response to brentuximab vedotin can occur in patients who have low or undetectable expression of CD30 (< 10%) [94]; this is being assessed in an ongoing phase II trial of brentuximab vedotin plus CHP in patients with PTCL and low CD30 expression (1–10%) [83].

## CD30-targeted therapy for CTCL

Like PTCL, CD30 expression in CTCL is variable by subtype [3]. CD30 is expressed in primary cutaneous ALCL (C-ALCL) and most cases of lymphomatoid papulosis [3]. Approximately 32% of classic MF and 59% of transformed MF cases are CD30-positive [3]. Rare CTCL subtypes including subcutaneous panniculitis-like T-cell lymphomas, aggressive epidermotropic cytotoxic T-cell lymphomas, and NK T-cell lymphomas, among others, have also been shown to express CD30 to varying degrees [3, 95]. Brentuximab vedotin is therefore indicated in Europe for the treatment of patients with CD30-positive CTCL who have received prior systemic therapy and in the USA for patients with C-ALCL and CD30-positive MF who have received prior systemic therapy [6, 7] (although the exact cut-off point for determination of CD30-positivity for most CTCL is still a matter of debate [3]). NCCN recommends brentuximab vedotin as a preferred systemic therapy option for MF (≥ stage IB–IIA or stage IV non-Sézary/visceral organ disease); brentuximab vedotin is also included as an option for Sézary Syndrome (SS) [80].

Key evidence for the efficacy of brentuximab vedotin in CTCL comes from the multicenter phase III ALCANZA study, which enrolled adults with CD30-positive MF or C-ALCL who had received at least 1 previous therapy [96]. Patients with high circulating Sézary cells (B2 level) were not included. Participants were randomized to receive brentuximab vedotin or physician’s choice of conventional therapy (oral methotrexate or bexarotene) [96]. At a median follow-up of 22.9 months, the proportion of patients with an overall response lasting at least 4 months (ORR4) was greater in the brentuximab vedotin arm compared with the physician’s choice arm (between-arm difference 43.8%, 95% CI 29.1–58.4; *p* < 0.0001), a difference that was observable in both the MF and C-ALCL subgroups [96]. Importantly, this was regardless of large-cell transformation (LCT) status and CD30 level at baseline in the MF subgroup [97]. Median PFS and EFS were also longer in the brentuximab vedotin arm versus the physician’s choice arm [96], and brentuximab vedotin treatment reduced symptom burden without impacting quality of life, as measured using the Skindex-29, Functional Assessment of Cancer Therapy-General and European QoL 5-dimension questionnaires [98]. AE rates with brentuximab vedotin were consistent with the known safety profile of the drug, and there were no new or unexpected toxicities [96]. PN was reported in 67% of patients receiving brentuximab vedotin, but this resolved or improved in most cases after completion or cessation of treatment [96]. Final data from the study, at a median follow-up of 45.9 months, have demonstrated significant and sustained benefits of brentuximab vedotin in terms of ORR4 and CR, as well as benefits to PFS for a median follow-up of 36.8 months [99].

## Real-world data on brentuximab vedotin in CTCL

Real-world studies on brentuximab vedotin therapy for CTCL support the results of the ALCANZA trial, with good responses to treatment observed in patients with MF and C-ALCL [100–104] and provide evidence for potential benefits of brentuximab vedotin in other CTCL subtypes [100, 104–107]. A European retrospective study in patients with MF and SS did not detect any differences in response by CD30 level or previous treatment, but suggested a potential association between lower disease stage and higher ORR4, as well as between LCT and skin ORR [102]. Conversely, a retrospective review of patients with MF and LCT in the USA detected superior outcomes with brentuximab vedotin for both early and late LCT disease (5-year OS of 61% and 83%, respectively) [108]. In a retrospective analysis of patients with relapsed or refractory SS, a patient group that was excluded from ALCANZA, 38% (5/13) experienced a global response, 23% (3/13) achieved ORR4, and 1 patient had a CR [105]. The aforementioned European study in patients with MF and SS did not detect any difference in treatment response between these two subtypes, suggesting that patients with SS can benefit from brentuximab vedotin treatment to a similar extent as MF patients [102]. Cases studies in patients with refractory primary cutaneous gamma-delta T-cell lymphoma have also noted benefits of brentuximab vedotin treatment [106, 107], and some retrospective studies of brentuximab vedotin for CTCL have included a range of different rare subtypes, noting treatment responses in folliculotropic MF, SS with LCT, and granulomatous MF [100, 104].

## Ongoing controversies for anti-CD30 therapy in CTCL

Of the 3 types of lymphoma discussed so far, brentuximab vedotin therapy for CTCL is the least well studied. While the benefits of brentuximab vedotin in MF and C-ALCL are well-established, there remains a need for assessments of the value of combining it with chemotherapy for C-ALCL, as well as further studies on other subtypes. Additionally, as for PTCL, data on response to brentuximab vedotin in patients with low or no CD30 expression are also required to determine the optimal role of brentuximab vedotin across CTCL subtypes.

## Targeting CD30 in other lymphomas

In addition to cHL, PTCL, and CTCL, there are other tumor types that express CD30 and may benefit from CD30-targeted therapy. For example, CD30 is frequently expressed by Epstein-Barr virus (EBV) positive lymphomas. In a phase II trial of brentuximab vedotin in patients with relapsed/refractory EBV-positive CD30-positive mature T, NK, or B-cell lymphoma, ORR was 48%, and at a median of 20 months follow-up, median PFS and OS were 6.2 months and 15.7 months, respectively [109]. Another CD30-expressing tumor type is post-transplant lymphoproliferative disorder, which is a rare complication of transplantation [110]. Up to 70% of these can be CD30-positive, suggesting a potential role for brentuximab vedotin in the treatment of this disorder [110].

Some CD30-positive B-cell specific lymphomas may also respond to brentuximab vedotin therapy. Although brentuximab vedotin has limited activity as a single agent in relapsed primary mediastinal B-cell lymphoma (PMBCL) [111], when used in combination with nivolumab in the phase I/II CheckMate 436 study, investigator-assessed ORR and complete remission rates of 73% and 37%, respectively, were achieved with a median follow-up of 11.1 months [112]. Brentuximab vedotin in combination with rituximab and CHP (with or without consolidative radiation) has been shown to be an active first-line regimen for PMBCL, DLBCL, and gray-zone lymphoma, in a phase I/II trial [113].

CD30 is variably expressed in DLBCL, at a frequency of up to 60% depending on cut-off value [4, 114], but unexpectedly, some studies have suggested that the response to brentuximab vedotin treatment does not correlate with CD30 expression [115]. This has led to a hypothesis that CD30-positive extracellular vesicles can transport brentuximab vedotin to CD30-negative tumor cells (a theory that is borne out by preclinical data) [115]. Finally, primary effusion lymphoma, a rare and aggressive large B-cell lymphoma prevalent in people living with HIV and occurring less commonly in non-HIV immunocompromised people, can be CD30-positive and has been shown to respond to second-line brentuximab vedotin therapy in a single case report [116].

## Conclusions and future perspectives

The last 10 years of experience with brentuximab vedotin have clearly demonstrated that CD30 is a highly valuable therapeutic target for lymphoma therapy. Exploitation of this target can improve outcomes for patients with a range of different CD30-positive lymphomas, and brentuximab vedotin is now an established standard of care in multiple treatment settings. Recent long-term data from phase III studies have demonstrated the durability of its benefit, and real-world data show that the benefits seen in clinical trials are translatable to clinical practice, including for tumor types not included in the trials. Several questions remain unanswered, however, including the importance of novel brentuximab vedotin combination regimens, such as those including checkpoint inhibitors. With respect to T-cell lymphoma, single-agent brentuximab vedotin is highly efficacious for the cutaneous lymphomas, ALCL and mycosis fungoides, even when they express low levels of CD30. The addition of chemotherapy to brentuximab vedotin in such groups requires further study. For patients with PTCL, the value of adding brentuximab vedotin to front-line chemotherapy for subtypes beyond systemic ALCL requires further study. In general, there remains a need for more data on brentuximab vedotin treatment of rare lymphoma subtypes, PTCL with low CD30 expression, and high-risk patient populations, at early disease stages, and in combination with checkpoint inhibitors.

The novel anti-CD30 therapies that are in development may be expected to further improve outcomes for patients with CD30-positive lymphomas in the coming years. Bispecific antibodies and CAR-T cell therapies have shown considerable promise in early studies in cHL and, with continued success, may be expanded to other CD30-positive lymphoma types. Innovations in CAR-T cell design to improve antitumor activity, such as incorporation of variable fragments targeting membrane-proximal epitopes or with human antigen-recognition domains, or use of memory stem T-cells, may help to further improve the efficacy of this therapy for CD30-positive tumors [117, 118].

Brentuximab vedotin has been a pioneer for the ADC approach to therapy. In the future, ADCs with novel antigen targets may also contribute to improved patient outcomes in lymphoma. Polatuzumab vedotin, an anti-CD79 ADC recently approved for the treatment of relapsed/refractory DLBCL, and camidanlumab tesirine, an anti-CD25 ADC in development for the treatment of various malignancies including cHL, have the potential to widen the range of treatment options for lymphoma [119, 120]. Advances in bioconjugation technologies to increase stability and homogeneity of ADCs may also contribute to improved ADC approaches in the future [121].
